# Inhibition of EGFR Signaling Protects from Mucormycosis

**DOI:** 10.1128/mBio.01384-18

**Published:** 2018-08-14

**Authors:** Tonya N. Watkins, Teclegiorgis Gebremariam, Marc Swidergall, Amol C. Shetty, Karen T. Graf, Abdullah Alqarihi, Sondus Alkhazraji, Abrar I. Alsaadi, Vonetta L. Edwards, Scott G. Filler, Ashraf S. Ibrahim, Vincent M. Bruno

**Affiliations:** a Institute for Genome Sciences, University of Maryland School of Medicine, Baltimore, Maryland, USA; b Division of Infectious Diseases, Los Angeles Biomedical Research Institute at Harbor, UCLA Medical Center, Torrance, California, USA; c David Geffen School of Medicine at UCLA, Torrance, California, USA; d Department of Microbiology and Immunology, University of Maryland School of Medicine, Baltimore, Maryland, USA; University of Maine; University of Texas Health Science Center

**Keywords:** EGFR, gefitinib, *Rhizopus*, mucormycosis

## Abstract

Mucormycosis is a life-threatening, invasive fungal infection that is caused by various species belonging to the order Mucorales. *Rhizopus* species are the most common cause of the disease, responsible for approximately 70% of all cases of mucormycosis. During pulmonary mucormycosis, inhaled *Rhizopus* spores must adhere to and invade airway epithelial cells in order to establish infection. The molecular mechanisms that govern this interaction are poorly understood. We performed an unbiased survey of the host transcriptional response during early stages of Rhizopus arrhizus var. delemar (R. delemar) infection in a murine model of pulmonary mucormycosis using transcriptome sequencing (RNA-seq). Network analysis revealed activation of the host’s epidermal growth factor receptor (EGFR) signaling. Consistent with the RNA-seq results, EGFR became phosphorylated upon *in vitro* infection of human alveolar epithelial cells with several members of the Mucorales, and this phosphorylated, activated form of EGFR colocalized with R. delemar spores. Inhibition of EGFR signaling with cetuximab or gefitinib, specific FDA-approved inhibitors of EGFR, significantly reduced the ability of R. delemar to invade and damage airway epithelial cells. Furthermore, gefitinib treatment significantly prolonged survival of mice with pulmonary mucormycosis, reduced tissue fungal burden, and attenuated the activation of EGFR in response to pulmonary mucormycosis. These results indicate EGFR represents a novel host target to block invasion of alveolar epithelial cells by R. delemar, and inhibition of EGFR signaling provides a novel approach for treating mucormycosis by repurposing an FDA-approved drug.

## IMPORTANCE

Mucormycosis is an increasingly common, highly lethal fungal infection with very limited treatment options. Using a combination of *in vivo* animal models, transcriptomics, cell biology, and pharmacological approaches, we have demonstrated that Mucorales fungi activate EGFR signaling to induce fungal uptake into airway epithelial cells. Inhibition of EGFR signaling with existing FDA-approved drugs significantly increased survival following R. arrhizus var. delemar infection in mice. This study enhances our understanding of how Mucorales fungi invade host cells during the establishment of pulmonary mucormycosis and provides a proof-of-concept for the repurposing of FDA-approved drugs that target EGFR function.

## INTRODUCTION

Mucormycosis is an invasive fungal infection of humans caused by species of the order Mucorales, subphylum Mucormycotina (1, 2). *Rhizopus* spp. are the most common etiologic agent of mucormycosis and are responsible for approximately 70% of all cases (1–3). The primary risk factors for mucormycosis include neutropenia, diabetes mellitus resulting in hyperglycemia and diabetic ketoacidosis (DKA), solid organ or bone marrow transplantation, treatment with corticosteroids, deferoxamine therapy, trauma and burns (e.g., wounded soldiers in combat), and malignant hematological disorders (2, 4).

The most common forms of mucormycosis, based on anatomical site, are rhino-orbital/cerebral, pulmonary, cutaneous, gastrointestinal, and disseminated. Rhino-orbital/cerebral mucormycosis is found almost exclusively in DKA patients, while pulmonary disease is mainly found in neutropenic patients (5). Cutaneous necrotizing mucormycosis outbreaks in healthy individuals have also been reported and often follow natural disasters or severe trauma (e.g., infections following the tsunami that devastated Southeast Asia in 2004 and the tornadoes that occurred in Joplin, MO, in June 2011) (6, 7). Since there are no federal requirements to report fungal infections, the true prevalence of mucormycosis is likely to be much higher than currently reported.

There are very few antifungal agents approved by the United States FDA for treating mucormycosis; the first is amphotericin B (AmB), which has been used to treat mucormycosis for the last 6 decades. AmB has significant nephrotoxicity, other adverse effects, and very limited clinical success (2, 8). Although isavuconazole and posaconazole have been recently approved to treat mucormycosis, neither is considered to be superior to AmB treatment (8, 9). In the absence of surgical removal of the infected focus (for example, excision of the eye in patients with rhinocerebral mucormycosis), antifungal therapy alone is rarely curative. Even when surgical debridement is combined with high-dose antifungal therapy, the mortality associated with mucormycosis is >50%. In patients with prolonged neutropenia or disseminated disease, mortality is 90 to 100% (10, 11). The limited treatment options coupled with the high mortality and morbidity rates and the frequently disfiguring surgical therapy provide a clear mandate to explore alternative approaches to treat this infection.

In the case of pulmonary mucormycosis, infection is generally acquired by inhalation of spores that are ubiquitous in nature. As lung epithelial cells are among the first host cells that interact with Mucorales spores during pulmonary infection, a molecular understanding of how these cells sense and respond to the pathogen is essential to understanding the pathogenesis of pulmonary mucormycosis. While the invasion of endothelial cells by Rhizopus arrhizus var. delemar (referred to from here on as R. delemar) is known to be mediated by the interaction between the fungus-encoded CotH3 and the host-encoded GRP78 (12, 13), the specific interactions that govern adherence and invasion of lung epithelial cells are poorly understood.

In this study, we used transcriptome sequencing (RNA-seq) to examine the host transcriptional response to R. delemar infection in a well-established *in vivo* murine model of pulmonary mucormycosis. Network analysis of the data set revealed the modulation of host pathways that were not previously linked to the host response to mucormycosis. We provide evidence that the epidermal growth factor receptor (EGFR) pathway is activated by R. delemar and other Mucorales infection and that this activation mediates invasion of lung epithelial cells by these fungi. Importantly, gefitinib, an FDA-approved drug that inhibits EGFR, protected mice from mucormycosis. These results suggest that inhibition of EGFR signaling provides a novel therapeutic approach for treating mucormycosis.

## RESULTS

### RNA-seq analysis of a murine model of mucormycosis.

We analyzed the host transcriptional response to R. delemar in a DKA murine model of pulmonary mucormycosis due to R. delemar infection. At 14 and 24 h postinoculation, animals were sacrificed, and the lungs were harvested for extraction of total RNA for subsequent transcriptome analysis using RNA-seq. These time points were chosen because they represent early stages of infection, prior to the onset of massive tissue damage and necrosis, which might complicate interpretation of transcriptome analyses. The early time points also allowed us to focus on the initial response of the lung tissue during adhesion of spores to and subsequent invasion of the airway epithelium. From each of the 12 sequencing libraries, we obtained an average of 96.4 ± 15.8 million reads that mapped to the mouse reference genome (see Table S1 in the supplemental material). The inclusion of a poly(A) enrichment step in the RNA-seq protocol, as predicted, resulted in the detection of transcripts from the infecting fungus. However, a robust analysis of the R. delemar transcriptome was precluded by the lack of sufficient reads that mapped to the R. delemar reference genome (6,488 reads combined from all six infected samples). Therefore, we focused our analysis of these samples on the host response.

10.1128/mBio.01384-18.5 TABLE S1  RNA-seq mapping statistics for each sample. Download TABLE S1, XLSX file, 0.1 MB. Copyright © 2018 Watkins et al. 2018 Watkins et al. This content is distributed under the terms of the Creative Commons Attribution 4.0 International license.

We have previously demonstrated that infection-induced transcriptome changes can be used to identify signaling pathways that govern the interaction between host and fungal pathogen (14–16). Using the Ingenuity Pathway Analysis (IPA) software (Ingenuity Systems; http://www.ingenuity.com), we performed an upstream regulator analysis on the sets of host genes that were differentially expressed (*P* < 0.05) (see Tables S2 and S3 in the supplemental material) between the infection groups and the appropriate time-matched negative-control groups, which were immunosuppressed but not infected.

10.1128/mBio.01384-18.6 TABLE S2  Differentially expressed host genes after 14 h of infection. Download TABLE S2, XLSX file, 0.2 MB. Copyright © 2018 Watkins et al. 2018 Watkins et al. This content is distributed under the terms of the Creative Commons Attribution 4.0 International license.

10.1128/mBio.01384-18.7 TABLE S3  Differentially expressed host genes after 24 h of infection. Download TABLE S3, XLSX file, 0.2 MB. Copyright © 2018 Watkins et al. 2018 Watkins et al. This content is distributed under the terms of the Creative Commons Attribution 4.0 International license.

**FIG 1:**
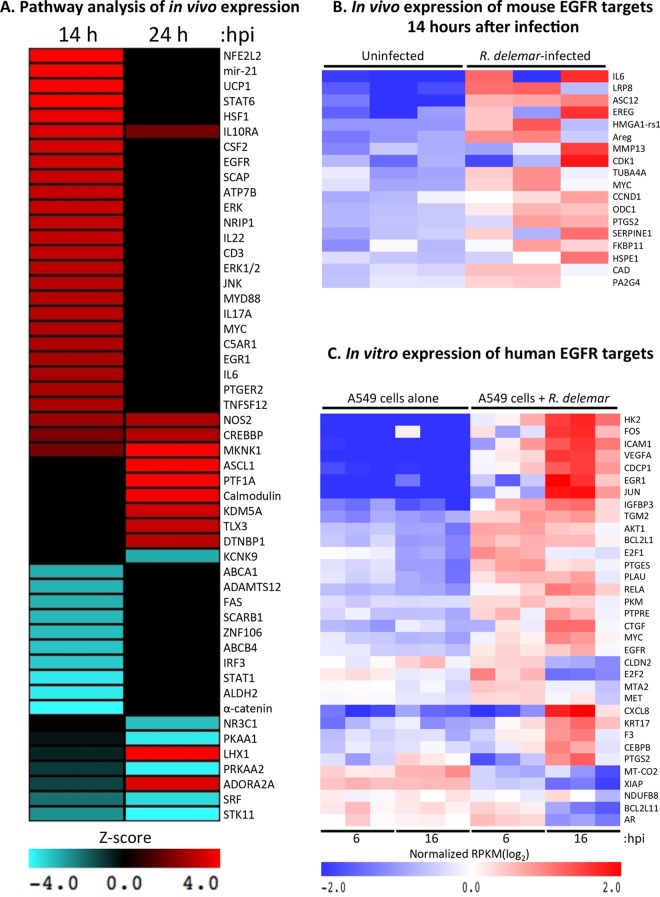
Host response to R. delemar infection *in vivo* and *in vitro*. (A) Mouse upstream regulators that are predicted to be changed in R. delemar-infected lungs in a mouse DKA model of mucormycosis. Red indicates predicted activation (*Z* score of >2). Teal indicates predicted repression (*Z* score of < −2). Black indicates no predicted effect. (B) Expression of known mouse EGFR targets in lungs 14 h postinoculation of the *in vivo* DKA model. (C) Expression of known human EGFR targets in A549 cells at 6 and 16 h following inoculation of an *in vitro* infection. Plotted for panels B and C are the log-transformed reads per kilobase per million (RPKM) values that have been normalized across all samples. Red indicates high gene expression; blue indicates low expression. Each column in panels B and C represents an individual sample from a different mouse.

This approach was validated by our identification of two pathways that were already linked to infection by Mucorales: interleukin-22 (IL-22) and IL-17A (Fig. 1A) (17). Our analysis also predicted the activation of signaling pathways that have not been tied to mucormycosis but have been associated with the host response to fungal infection, including colony-stimulating factor 2 (CSF2), extracellular signal-regulated kinases (ERKs), myeloid differentiation primary response 88 (MYD88), and JNK (Jun N-terminal kinase) (18–24) (Fig. 1A). We also noticed a striking temporal dynamic in our data set. Specifically, the majority of the pathways are modulated at 14 h postinoculation (hpi) and returning to normal 24 h postinfection. Furthermore, a smaller subset of pathways was modulated only at 24 h postinfection.

Of particular interest was the significant overlap between genes that are differentially expressed 14 h postinoculation with R. delemar and the known transcriptional targets of the epidermal growth factor receptor (EGFR) signaling pathway (*P* value of overlap, 9.57 × 10^−6^). Specifically, R. delemar infection induced the expression of 18 genes that are known to be activated by EGFR signaling (Fig. 1B), providing evidence for the activation of EGFR protein in response to R. delemar infection.

Further support of the activation of EGFR signaling was provided by the predicted activation of mir-21 (*P* value overlap, 8.6 × 10^−15^ [Fig. 1A]), a microRNA that governs the expression of genes involved in many different biological processes (25–30). EGFR activation enhances the expression of mir-21 in lung epithelial cells (31). Our sequencing approach, which is geared toward the detection of long transcripts, does not allow the examination of microRNAs. However, in our infection model, 28 known repression targets and 5 known activation targets of mir-21 were downregulated or upregulated, respectively, 14 h after inoculation relative to the uninfected control group (see Table S4 in the supplemental material). This downregulation of mir-21-repressed genes is consistent with an EGFR-stimulated increase in mir-21 expression. In the database used to perform the upstream regulator analysis, none of the 33 mir-21 target genes are annotated as EGFR targets, and they are therefore not included in Fig. 1C, so the total number of differentially expressed genes that provide evidence of EGFR pathway activation is 51.

10.1128/mBio.01384-18.8 TABLE S4  Known mir-21 target genes that are differentially expressed after 14 h of infection. Download TABLE S4, XLSX file, 0.1 MB. Copyright © 2018 Watkins et al. 2018 Watkins et al. This content is distributed under the terms of the Creative Commons Attribution 4.0 International license.

### EGFR signaling is activated during *in vitro* infection of airway epithelial cells.

We have previously examined the transcriptional response of A549 human alveolar epithelial cells to infection with R. delemar at 6 and 16 h (15). Upstream regulator analysis of this *in vitro* RNA-seq data set also revealed a significant overlap between genes that are differentially expressed following R. delemar infection and the known transcriptional targets of the EGFR signaling pathway (*P* values of 4.33 × 10^−2^ and 1.43 × 10^−3^ for 6 and 16 h, respectively). Specifically, R. delemar infection induced changes in gene expression of 34 known downstream targets of EGFR signaling in a direction that is consistent with the activation of the EGFR (29 activated and 5 repressed [Fig. 1C]).

**FIG 2:**
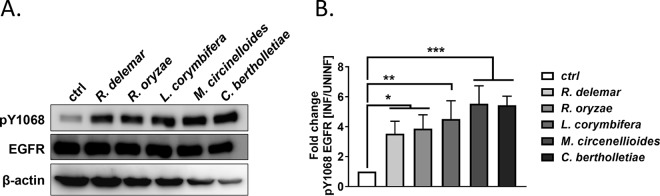
Phosphorylation and localization of EGFR in A549 cells infected with Mucorales. (A) Representative immunoblot examining tyrosine phosphorylation of EGFR residue Y1068 in response to individual infection with five different Mucorales fungi. (B) Densitometric analysis of the immunoblots. ctrl, control.

**FIG 3:**
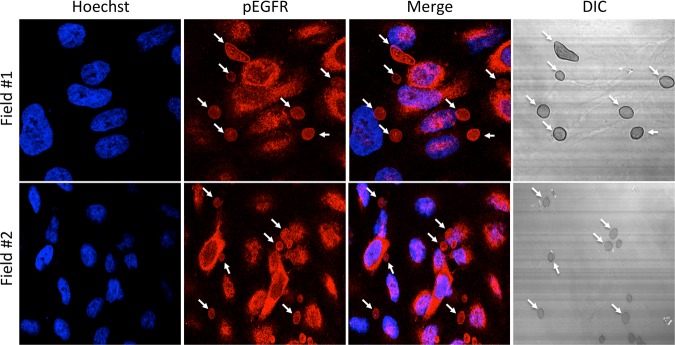
Localization of phosphor-EGFR in A549 cells infected with R. delemar. A549 cells were infected for 30 min with 2 × 10^5^ R. delemar spores that had been germinated for 1 h. Cells were then stained for phospho-EGFR (red) and host cell nuclei using Hoechst (blue). DIC, differential inference contrast.

When EGFR is activated by ligand binding, tyrosine residues at the intracellular carboxy terminus become phosphorylated and the signal is transmitted to a variety of downstream signaling pathways (32). To confirm the EGFR activation by an orthogonal approach, we tested whether R. delemar infection of the A549 pulmonary epithelial cell line stimulated the phosphorylation of EGFR by immunoblotting. R. delemar infection induced the phosphorylation of EGFR on tyrosine residue 1068 when examined 3 h postinfection (Fig. 2). The same phosphorylation was also induced following infection with four other Mucorales species—Rhizopus oryzae, Lichtheimia corymbifera, Mucor circinelloides, and Cunninghamella bertholletiae (Fig. 2)—indicating that EGFR activation is not a strain- or species-specific phenomenon. Consistent with these results, we observed that the phosphorylated (activated) form of EFGR colocalized with R. delemar spores during *in vitro* infection of A549 cells (Fig. 3). We did not observe colocalization when R. delemar spores were examined in the absence of A549 cells (see Fig. S1 in the supplemental material). Taken together, these results are consistent with a model in which Mucorales interacts with EGFR and activates signaling in airway epithelial cells.

10.1128/mBio.01384-18.1 FIG S1  The anti-EGFR antibodies do not bind to R. delemar cells. R. delemar spores that had been germinated for 1 h were stained for pEGFR (red) and EGFR (green) in the absence of host cells. Download FIG S1, PDF file, 0.4 MB. Copyright © 2018 Watkins et al. 2018 Watkins et al. This content is distributed under the terms of the Creative Commons Attribution 4.0 International license.

### EGFR signaling governs the uptake of Mucorales and subsequent damage of airway epithelial cells.

**FIG 4:**
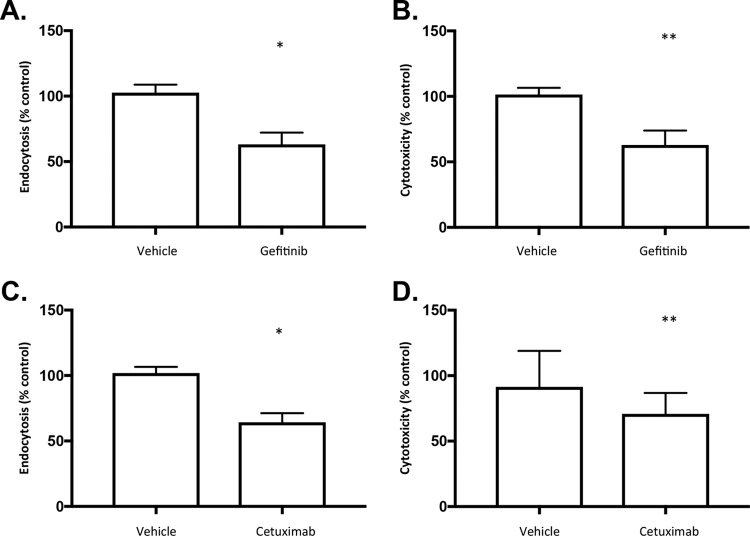
Effects of EGFR inhibition on invasion of airway epithelial cells by R. delemar. A549 alveolar epithelial cells were pretreated with vehicle, 25 µM gefitinib, or 25 µg/ml cetuximab for 1 h followed by (top) 3 h of infection in the presence of inhibitor with 2 × 10^5^ R. delemar spores or (bottom) 24 h of infection (in the presence of inhibitors) with 2 × 10^6^ R. delemar spores that were germinated for 1 h. *, *P* < 0.0001, and **, *P* < 0.01, versus control by Wilcoxon rank-sum test. Data are expressed as median ± interquartile range and represent at least two independent experiments.

The predicted activation of EGFR and mir-21 signaling early during the infection process, the colocalization of activated EGFR with R. delemar, and the involvement of EGFR in invasion by Candida albicans and diverse microbial pathogens (33–35) compelled us to explore the possibility that EGFR mediates the invasion of airway epithelial cells by R. delemar. Thus, we next tested whether blocking EGFR signaling would protect alveolar epithelial cells from invasion by R. delemar. We used gefitinib, a clinically relevant EGFR kinase inhibitor, to study its effect on R. delemar-mediated endocytosis by alveolar epithelial cells and their subsequent damage. When A549 cells were pretreated for 1 h with 25 µM gefitinib, endocytosis of R. delemar spores was significantly reduced compared to pretreatment with vehicle alone (Fig. 4A). Pretreatment with gefitinib also significantly reduced R. delemar-induced damage of A549 cells, when assayed by lactate dehydrogenase (LDH) assay (Fig. 4B).

**FIG 5:**
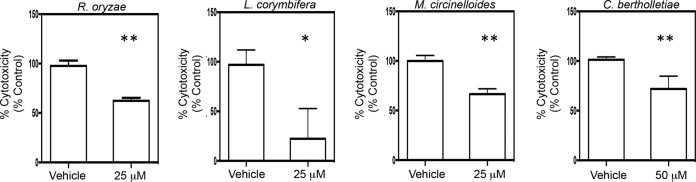
Gefitinib inhibits infection-induced damage of airway epithelial cells by four different species of Mucorales. A549 alveolar epithelial cells were pretreated with vehicle or gefitinib (25 or 50 µM) for 1 to 2 h followed by 24 h of infection (in the presence of inhibitors) with 2 × 10^6^ (C. bertholletiae and R. oryzae) or 3 × 10^6^ (L. corymbifera and M. circineollides) spores that were germinated for 1 h *, *P* < 0.0001, and **, *P* < 0.01, versus the control by Wilcoxon rank sum test. Data are expressed as median ± interquartile range and represent at least two independent experiments done in triplicate.

To complement our studies with gefitinib, which targets the intracellular tyrosine kinase domain of EGFR, we examined the ability of cetuximab to block epithelial cell invasion. Cetuximab is a monoclonal antibody that recognizes the extracellular portion of EGFR and has been shown to block ligand-dependent activation of EGFR signaling (36). When the same host cells were pretreated for 1 h with 25 µg/ml cetuximab, endocytosis of R. delemar spores and host cell damage were both significantly reduced compared to pretreatment with an equivalent amount of IgG1 control antibody (Fig. 4C and D). Notably, neither gefitinib nor cetuximab inhibited R. delemar mycelial growth (see Fig. S2 in the supplemental material). Pretreatment of R. delemar spore preparations with gefitinib or cetuximab, followed by rinsing, prior to the infection did not reduce endocytosis or host cell damage (see Fig. S3 in the supplemental material). The concordance between the results of the endocytosis assay and the host cell damage assay is consistent with previous observations that endocytosis of R. delemar is a prerequisite for inducing host cell damage of endothelial cells (37). We also found that pretreatment with gefitinib reduced fungus-induced damage of A549 cells following infection with R. oryzae, L. corymbifera, M. circinelloides, and C. bertholletiae (Fig. 5). Collectively, these results indicate that EGFR signaling is required for maximal invasion of and damage to alveolar epithelial cells by Mucorales.

10.1128/mBio.01384-18.2 FIG S2  EGFR inhibitors do not inhibit hyphal growth of R. delemar. R. delemar spores were incubated in F-12K medium plus 10% FBS in the presence of DMSO, 25 µM gefitinib, 25 µg/ml IgG, or 25 µg/ml cetuximab in tissue culture dishes without shaking at 37°C in 5% CO_2_. Download FIG S2, PDF file, 38.1 MB. Copyright © 2018 Watkins et al. 2018 Watkins et al. This content is distributed under the terms of the Creative Commons Attribution 4.0 International license.

10.1128/mBio.01384-18.3 FIG S3  Effects of R. delemar pretreatment with EGFR inhibitors on internalization of A549 cells. R. delemar spores were pretreated with 25 µM gefitinib or 25 µg/ml cetuximab for 1 h followed by washing with F-12K plus 10% FBS medium. A549 alveolar epithelial cells were then infected with 2 × 10^5^ R. delemar spores for 3 h. The results of treatment versus control were compared by Wilcoxon rank sum test. Data are expressed as median ± interquartile range. Download FIG S3, PDF file, 0.1 MB. Copyright © 2018 Watkins et al. 2018 Watkins et al. This content is distributed under the terms of the Creative Commons Attribution 4.0 International license.

EGFR signaling has also been shown to facilitate invasion of oral epithelial cells by C. albicans which, like R. delemar, enters cells by induced endocytosis. The EGFR-dependent invasion of C. albicans requires the activation of the aryl hydrocarbon receptor (Ahr) and the subsequent activation of Src family kinases, which in turn phosphorylate and activate EGFR (34, 38). To address whether the same mechanism is being employed to facilitate invasion of airway epithelial cells by R. delemar, we measured the effect of an Ahr inhibitor (CH-223191) and an Src inhibitor (Src kinase inhibitor II) on endocytosis. Neither of the inhibitors altered the ability of A549 cells to endocytose R. delemar spores (see Fig. S4 in the supplemental material). These results suggest that the mechanism of activation of EGFR in epithelial cells is distinct from the activation of EGFR by C. albicans.

10.1128/mBio.01384-18.4 FIG S4  Effects of Ahr and Src inhibition on R. delemar internalization and damage. A549 alveolar epithelial cells were pretreated with 10 µM CH-223191 or 10 µM Src inhibitor for 1 h followed by 3 h of infection with 2 × 10^5^ R. delemar spores that were germinated for 1 h. *P* < 0.05 for control versus treatment by Wilcoxon rank sum test. Data are expressed as median ± interquartile range. ns, not significant. Download FIG S4, PDF file, 0.1 MB. Copyright © 2018 Watkins et al. 2018 Watkins et al. This content is distributed under the terms of the Creative Commons Attribution 4.0 International license.

### Gefitinib treatment increases survival of mice with mucormycosis.

**FIG 6:**
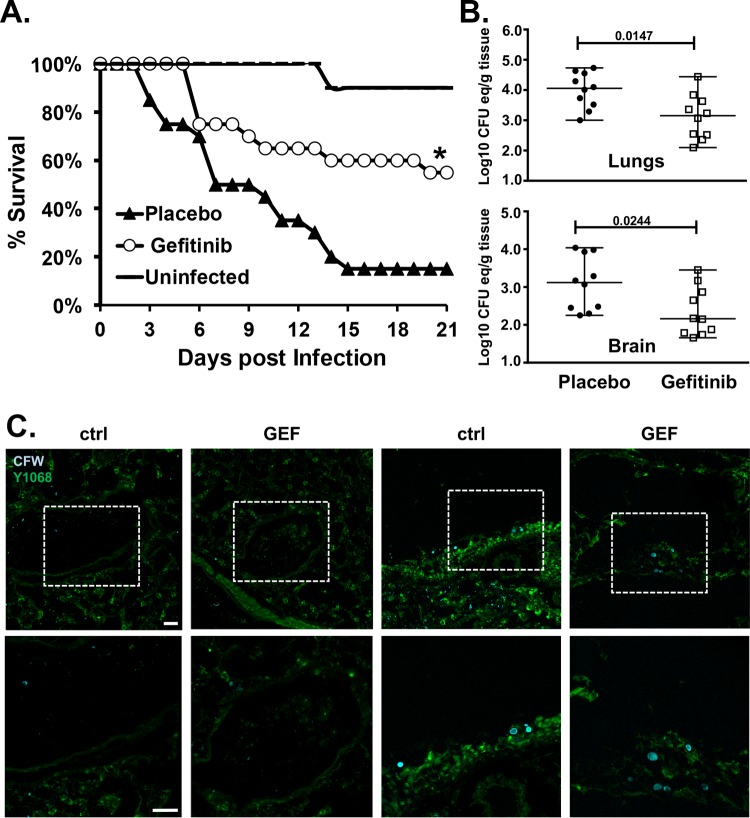
Gefitinib inhibits EGFR phosphorylation *in vivo* and protects mice from pulmonary mucormycosis. (A) Survival of neutropenic mice (*n =* 20/group from two independent experiments with similar results) infected intratracheally with R. delemar (average inoculum of 3.8 × 10^3^ spores per mouse) and treated with vehicle control (placebo) or 10 mg/kg gefitinib 4 h postinfection for 5 consecutive days. *, *P* = 0.0084 versus placebo-treated mice by log rank test. (B) Tissue fungal burden of lungs and brains of mice (*n =* 10 per group) infected intratracheally with R. delemar (5.6 × 10^3^ spores per mouse of confirmed inoculum) and treated with vehicle control (placebo) or gefitinib. Data are presented as median ± interquartile range. (C) Twenty-four hours post-intratracheal infection, lungs of gefitinib (GEF)-treated or untreated mice were harvested, sectioned, and stained with calcofluor white (CFW) and anti-pY1068 antibody. Scale bars, 20 µm. Dashed boxes in the top row of panels indicate the sections shown enlarged in the bottom row. ctrl, control.

We next sought to determine if EGFR signaling governs the establishment and/or progression of mucormycosis in a well-established *in vivo* murine model of mucormycosis. Unfortunately, mice harboring deletions in EGFR die within the first 8 days of life (39), thus precluding our ability to test the receptor in a traditional mouse gene deletion experiment. Furthermore, there are no published lung-specific deletion models for EGFR. Therefore, we infected neutropenic mice intratracheally with R. delemar (40) and treated them with 10 mg/kg of body weight gefitinib (41) or vehicle alone (placebo) for 5 consecutive days starting 4 h postinfection. We chose to begin the intervention at 4 h because invasion of lung epithelial cells is an early event in the infection. Placebo-treated mice had a median survival time of 8 days and 85% mortality by day 15 postinfection (Fig. 6A). In contrast, mice treated with gefitinib had a median survival time of >21 days, and 55% of the mice remained alive by day 21, when the experiment was terminated, and the surviving mice appearing healthy. Corroborating the effects of gefitinib on survival, mice treated with this drug had an ~1-log reduction in their lungs and brains relative to placebo-treated mice (Fig. 6B). Consistent with these results, we found that infection with R. delemar induced marked tyrosine phosphorylation of EGFR in the lung (Fig. 6C) and that treatment of mice with gefitinib significantly reduced this phosphorylation (Fig. 6C). These results validate our *in vitro* observations and support the potential of targeting EGFR signaling as a novel therapeutic strategy for mucormycosis by immediately repurposing currently FDA-approved cancer drugs.

## DISCUSSION

Invasion of the airway epithelium is a crucial, yet poorly understood, step in the initiation of pulmonary mucormycosis. We took an unbiased approach to understand the host side of this interaction by performing RNA-seq analysis of infected lungs harvested during early stages of a well-established clinically relevant murine model of mucormycosis. A network analysis of differential gene expression in these mice revealed the enrichment for genes that are known to be downstream of EGFR and its regulatory target, mir-21. Because our analysis was performed on RNA harvested from whole lungs of infected mice, we were unable to determine which cell types responded to the infection with the increased gene expression. The predicted activation of EGFR signaling at 14 h, and not 24 h, could be indicative of a host response to the early stage of infection when R. delemar first invades the airway epithelium. Our *in vitro* analyses of human airway epithelial (A549) cells support this idea. Specifically, 34 EGFR-responsive genes are modulated during infection with R. delemar, and phospho-EGFR colocalized with endocytosed spores. Furthermore, pharmacological inhibition of EGFR signaling reduced both fungus-induced cytotoxicity and endocytosis of the spores by the A549 cells. Collectively, these results suggest that EGFR signaling mediates the invasion of airway epithelial cells by R. delemar. Hence, EGFR is the first host cell receptor to be implicated in the interaction between R. delemar and airway epithelial cells.

In general, EGFR signaling can be activated by binding to any one of seven known host-derived ligands, all of which are expressed as precursor proteins that span the plasma membrane and are cleaved by cell surface proteases upon stimulation (42). Upon cleavage, the extracellular domains are released as mature proteins into the extracellular space, where they can bind to and activate EGFR on the surface of the same cells or on adjacent cells (42). In addition, many pathogens, including viruses, bacteria, and fungi, seem to have evolved the ability to exploit EGFR signaling to gain entry into host cells (33), and the mechanism of EGFR activation across these pathogenic microbes varies. For example, in the context of viral infection, hepatitis C virus increases signaling by disrupting EGFR recycling to enhance its surface expression (43, 44). In contrast, the Campylobacter jejuni bacterium induces lipid raft formation, resulting in clustering and subsequent activation of EGFR (45), while meningitic Escherichia coli activates EGFR signaling by increasing sphingosine 1-phosphate (S1P_2_)-dependent release of heparin-binding ligand-like epidermal growth factor (HB-EGF) (35). The fungal pathogen C. albicans expresses two surface proteins, Als3p and Ssa1p, that likely bind to and activate EGFR and ErbB2 heterodimers (34).

At this point, we do not know the molecular mechanism by which EGFR signaling is being stimulated during R. delemar infection. The colocalization of EGFR with fungal spores is consistent with an interaction between a fungal cell wall component and EGFR. The invasion of A549 cells by an R. delemar strain with attenuated expression of CotH3 (12), the Mucorales ligand to host GRP78 during interaction with human umbilical vein endothelial cells, was not different from A549 cell invasion caused by wild-type R. delemar (Ashraf Ibrahim, unpublished data). Thus, CotH3 protein is not the fungal ligand by which EGFR is activated. Furthermore, the R. delemar genome does not encode a recognizable ortholog of *ALS3* or *SSA1* and inhibition of Src kinase activity or Ahr does not inhibit R. delemar invasion, suggesting that EGFR stimulation by Mucorales occurs via a different mechanism than that by C. albicans. Further experiments are required to determine the molecular nature of the activation.

Our *in vitro* blocking data using EGFR inhibitors did not entirely abrogate the ability of R. delemar to invade and damage alveolar epithelial cells. This strongly indicates that receptors other than EGFR are involved in the interaction of *Rhizopus* with these host cells. We have previously identified a host cell receptor, GRP78, required for R. delemar hematogenous dissemination. Specifically, the host 78-kDa glucose-regulated protein GRP78 interacts with the Mucorales-specific CotH3 cell wall protein during invasion of human umbilical vein endothelial cells (12). Because of the lack of CotH3 contribution to invasion of A549 cells (Ibrahim, unpublished), it is unlikely that GRP78 acts as a coreceptor with EGFR during interactions with invading Mucorales. Alternatively, GRP78 could contribute to Mucorales invasion of alveolar epithelial cells through interacting with a fungal ligand(s) other than CotH3. These possibilities are the topic of active investigation.

Since EGFR plays a central role in the progression of several types of cancer, significant effort has been put forth into developing therapies that target EGFR function (46). To this end, gefitinib, a small molecular inhibitor of EGFR kinase activity, and cetuximab, a monoclonal antibody specific for the extracellular domain of EGFR, provide valuable tools to test the role of EGFR in epithelial cell invasion by R. delemar. Indeed, in our mouse model of mucormycosis, treatment with gefitinib significantly increased survival and reduced tissue fungal burden of target organs. These results are consistent with a model in which R. delemar stimulates EGFR signaling *in vivo* to facilitate endocytosis of the fungus by airway epithelial cells and raise the exciting possibility of repurposing an FDA-approved drug to potentially control these invasive fungal infections. Since immunocompromised patients are the most susceptible and likely to develop mucormycosis, the observed efficacy of gefitinib in neutropenic mice adds to the clinical relevance and the potential for our results to translate to humans as an adjunctive therapy to current antifungal agents. Furthermore, because gefitinib targets the host and does not affect the growth or morphology of the fungus, acquisition of resistance is less likely to occur.

To summarize, we have identified host EGFR as a key mediator of early invasion of alveolar epithelial cells by Mucorales. Of great clinical importance is our finding that inhibition of EGFR function by an FDA-approved drug ameliorates murine mucormycosis and is likely to represent a new therapeutic modality as adjunctive therapy to lethal mucormycosis.

## MATERIALS AND METHODS

### Fungal strains and host cells.

R. delemar strain 99-880 (a clinical isolate obtained from a patient with rhino-orbital mucormycosis), R. oryzae strain 99-892, L. corymbifera strain 008-049, M. circinelloides strain NRRL3631, and C. bertholletiae strain 175 were grown on peptone-dextrose agar (PDA) plates for 3 to 5 days at 37°C. Spores were collected in endotoxin-free Dulbecco’s phosphate-buffered saline (DPBS), washed with endotoxin-free DPBS, and counted with a hemocytometer to prepare the final inocula. To form germlings, spores were incubated in yeast extract-peptone-dextrose (YPD) with shaking for 1 h at 37°C. Germlings were washed twice with endotoxin-free DPBS. The A549 type II pneumocyte cell line was grown in tissue culture dishes in F-12K medium with l-glutamine plus 10% fetal bovine serum (FBS).

### Drug.

Src kinase inhibitor II (CAS 459848-35-2; Calbiochem) was obtained from Millipore (catalog no. 567806).

### Murine models of mucormycosis.

For the *in vivo* RNA-seq experiments, diabetic ketoacidosis (DKA) was induced and mice were infected intratracheally with a target inoculum of 2.5 × 10^5^ fungal spores of R. delemar 99-880 in 25 µl as previously described (12). To test the effect of gefitinib on mouse survival following infection, male ICR mice (20 to 25 g [from Envigo]) were immunosuppressed by cyclophosphamide (200 mg/kg administered intraperitoneally [i.p.]) and cortisone acetate (500 mg/kg administered subcutaneously) given on days −2, +3, and + 9 relative to infection. This treatment resulted in 16 days of pancytopenia (40). To control for bacterial infection, immunosuppressed mice received 50 mg/liter enrofloxacin (Baytril; Bayer, Leverkusen, Germany) *ad libitum* on day −3 through day 0, after which the enrofloxacin was replaced with daily ceftazidime (5 mg/mouse) treatment administered subcutaneously through day +13 relative to infection. Mice were infected with 2.5 × 10^5^ spores of R. delemar 99-880 in 25 µl PBS given intratracheally as previously described (40). Treatment with gefitinib (10 mg/kg dissolved in 10% dimethylacetamide–90% polyethylene glycol 300 [DMA-PEG 300] and administered i.p.) started 4 h postinfection and continued once daily through day +4. Placebo-treated mice received DMA-PEG 300. Survival of mice served as the primary endpoint, with moribund mice humanely euthanized. To determine the effect of treatment on tissue fungal burden, mice were immunosuppressed and infected as described above. Gefitinib treatment started 4 h postinfection and continued through day +3. On day +4, mice were sacrificed, and lungs and brains, representing primary and secondary target organs (40), were collected and processed for tissue fungal burden by quantitative PCR (qPCR) (47). Values are expressed as log_10_ spore equivalents per gram of tissue.

All animal studies were approved by the Institutional Animal Care and Use Committee (IACUC) of the Los Angeles Biomedical Research Institute at Harbor-UCLA Medical Center according to the NIH guidelines for animal housing and care (approval reference no. 21125).

### Isolation of RNA from lung tissue.

Male ICR mice were immunosuppressed and infected as described above. Lungs were harvested 14 or 24 h postinfection and flash frozen in liquid nitrogen prior to extracting total RNA using Tri reagent solution (Ambion).

### RNA-seq and gene expression analysis.

Our deep sequencing analysis was performed in triplicate with three lungs per group isolated from 3 different mice. Sequencing libraries (non-strand-specific, paired end) were prepared with the TruSeq RNA sample prep kit (Illumina). The total RNA samples were subjected to poly(A) enrichment as part of the TruSeq protocol. One hundred fifty nucleotides of sequence was determined from both ends of each cDNA fragment using the HiSeq platform (Illumina) per the manufacturer’s protocol. Sequencing reads were annotated and aligned to the UCSC mouse reference genome (mm10, GRCm38.75) using TopHat2 (48). The alignment files from TopHat2 were used to generate read counts for each gene, and a statistical analysis of differential gene expression was performed using the edgeR package from Bioconductor (49). A gene was considered differentially expressed if the *P* value for differential expression was less than 0.05. To identify modulated signal transduction pathways, we used the upstream regulator analytic of IPA (Ingenuity Systems) to identify signaling proteins that are potentially activated or repressed during the course of infection. This analysis determines the overlap between lists of differentially expressed genes and an extensively curated database of regulator-target gene relationships. It then considers the direction of the gene expression changes to make predictions about activation or repression of specific pathways.

### Immunoblot of EGFR phosphorylation *in vitro*.

The A549 type II pneumocyte cell line (American Type Culture Collection) was grown as described previously (50). A549 cells in 24-well tissue culture plates were incubated in F-12K tissue culture medium supplemented with fetal bovine serum to a final concentration of 10%. Prior to infection, the A549 cells were serum starved for 120 min. Spores of R. delemar, R. oryzae, L. corymbifera, M. circinelloides, or C. bertholletiae were incubated in RPMI for 60 min at 37C, washed, and suspended in F-12K medium. A549 cells were infected for 3 h with a multiplicity of infection (MOI) of 5. Next, the cells were rinsed with cold Hanks balanced salt solution (HBSS) containing protease and phosphatase inhibitors and removed from the plate with a cell scraper. After collecting the cells by centrifugation, they were boiled in 2× SDS sample buffer. The lysates were separated by SDS-PAGE, and Y1068 EGFR phosphorylation was detected with a phospho-specific antibody (Cell Signaling; no. 2234). The blots were then stripped, and total protein levels and β-actin were detected by immunoblotting with appropriate antibodies against EGFR (Cell Signaling; no. 4267), and β-actin (Cell Signaling; 3700). The immunoblots were developed using enhanced chemiluminescence and imaged with a C400 (Azure Biosystems) digital imager.

### Measurement of R. delemar-induced host cell damage.

R. delemar-induced A549 cell damage was quantified using the Pierce LDH assay, with slight modifications to the manufacturer’s protocol. Briefly, A549 cells were grown in 96-well tissue culture plates for 18 to 24 h. Cells were then pretreated for 1 h with gefitinib (25 µM) or cetuximab (25 µg/ml) and infected with 2 × 10^6^ germlings suspended in 150 µl F-12K plus 10% FBS. For controls, host cells were incubated with dimethyl sulfoxide (DMSO) (the solvent used to reconstitute the inhibitor) or 25 µg/ml of mouse IgG antibody in parallel. After 24 h of incubation at 37°C, 50 µl of the cell culture supernatant was collected from uninfected, infected, and fungus-only control wells and transferred to a 96-well plate to assay for LDH activity. Lysis buffer was added to all infected wells, and the mixture was incubated for 45 min at 37°C. After lysis, 50 µl of cell culture supernatant was transferred to a 96-well plate and used for the LDH assay kit per the protocol. LDH release was calculated as follows: % cytotoxicity = [(experimental release − fungal cell spontaneous control − host cell spontaneous control)/(host cell maximum control − fungal cell maximum control − host cell spontaneous control)] × 100. LDH is a cytosolic enzyme but will be released into the cell culture medium upon cell membrane damage. The amount of extracellular LDH is proportional to the amount of cell damage.

### Measurement of host cell endocytosis.

Twelve-millimeter glass coverslips were seeded with A549 alveolar epithelial cells. Cells were then pretreated for 1 h with gefitinib (25 µM) or cetuximab (25 µg/ml). For controls, host cells will be incubated with DMSO (the solvent used to reconstitute the inhibitor) or 25 µg/ml mouse IgG antibody in parallel. Host cells were then infected with 2 × 10^5^ R. delemar spores. After incubation for 3 h, cells were fixed in 3% paraformaldehyde and stained for 1 h with 1% Uvitex, which specifically binds to chitin in the fungal cell wall. After being washed with PBS, coverslips were mounted on a glass slide with a drop of ProLong Gold antifade reagent (Molecular Probes) and sealed. The total number of cell-associated organisms (i.e., fungi adhering to the monolayer) per high-powered field was determined by phase-contrast microscopy. The same field was then examined by epifluorescence microscopy, and the number of brightly fluorescent, uninternalized fungi was determined. The number of endocytosed organisms was calculated by subtracting the number of fluorescent fungi from the total number of visible fungi. At least 400 organisms were counted per treatment group in at least 15 different fields per coverslip. Experiments were performed in duplicate or triplicate on at least two separate days.

### Confocal microscopy.

The accumulation of epithelial cell EGFR and pEGFR around R. delemar was visualized using the Zeiss LSM Duo confocal microscopy system. Twelve-millimeter glass coverslips in 12-well dishes were seeded with A549 alveolar epithelial cells and infected with 2 × 10^5^ R. delemar spores. After incubation at 37°C, cells were washed with HBSS and fixed with 3% paraformaldehyde. Cells were blocked and incubated with 1:500 mouse anti-EGFR (Santa Cruz; no. sc-373746) and 1:500 rabbit anti-pEGFR (Cell Signaling; no. 3777). Coverslips were washed and counterstained with 1:500 Alexa Fluor 546-labeled goat anti-mouse IgG and Alexa Fluor 488-labeled goat anti-rabbit IgG. Host cell nuclei were stained with Hoechst 33342 (Thermo Fisher). After washing, coverslips were mounted on a glass slide with ProLong Gold antifade reagent (Molecular Probes) and viewed by z-stacking using the Zeiss LSM Duo confocal microscopy system.

### Immunofluorescence of EGFR phosphorylation *in vivo*.

CD-1 mice were immunosuppressed with cyclophosphamide (200 mg/kg) and cortisone acetate (500 mg/kg) on day −2 relative to infection. They were inoculated by intratracheal injection of 1.0 × 10^7^ R. delemar cells. After 24 h of infection, the mice were sacrificed and the lungs were harvested, snap-frozen in optimal cutting temperature (OCT) compound. Ten-micrometer-thick sections were cut with a cryostat and fixed with cold acetone. Protein phosphorylation was detected as described elsewhere (38, 51). Briefly, the cryosections were rehydrated in PBS and then blocked with 10% bovine serum albumin (BSA). Sections were stained with a phospho-EGFR antibody (Cell Signaling; no. 2234), followed by a secondary antibody. The organisms were stained with calcofluor white (Sigma-Aldrich; no. 18909) and imaged by confocal microscopy.

### Statistical analyses.

*In vitro* experiments were performed in triplicate on two or three separate days. Data are expressed as the median ± interquartile range. Treatment groups were compared to controls using the Wilcoxon rank sum test. For the murine studies, survival of mice was analyzed using the log rank test, whereas differences in tissue fungal burden were analyzed by the Wilcoxon rank sum test using GraphPad Prism 6. *P* values of <0.05 were considered significant.

### Accession number(s).

All of the raw sequencing reads from this study have been submitted to the NCBI sequence read archive (SRA) under BioProject accession no. PRJNA429656 (https://www.ncbi.nlm.nih.gov/bioproject/PRJNA429656). The specific sample accession numbers are presented in Table S1.
