# Supplementary material for: Inhibition of EGFR Signaling Protects from Mucormycosis
Source: mBio. 2018 Aug 14;9(4):e01384-18. doi: 10.1128/mBio.01384-18 (PMC6094478; doi:10.1128/mBio.01384-18)
Supplement: FIG S1 [file mbo004184021sf1.pdf]

Supplementary Figure 1.

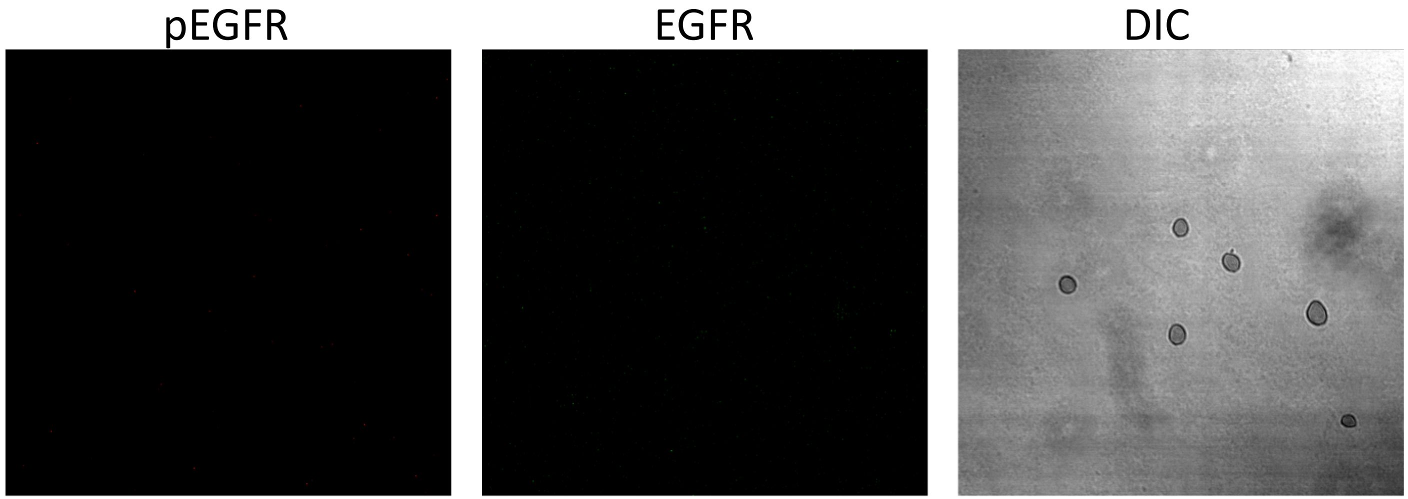

Supplementary Figure 1. The anti-EGFR antibodies do not bind to *R. delemar* cells. *R. delemar* spores that had been germinated 1 h were stained for pEGFR (red) and EGFR (green) in the absence of host cells.
