# Supplementary material for: Inhibition of EGFR Signaling Protects from Mucormycosis
Source: mBio. 2018 Aug 14;9(4):e01384-18. doi: 10.1128/mBio.01384-18 (PMC6094478; doi:10.1128/mBio.01384-18)
Supplement: FIG S2 [file mbo004184021sf2.pdf]

Supplementary Figure 2.

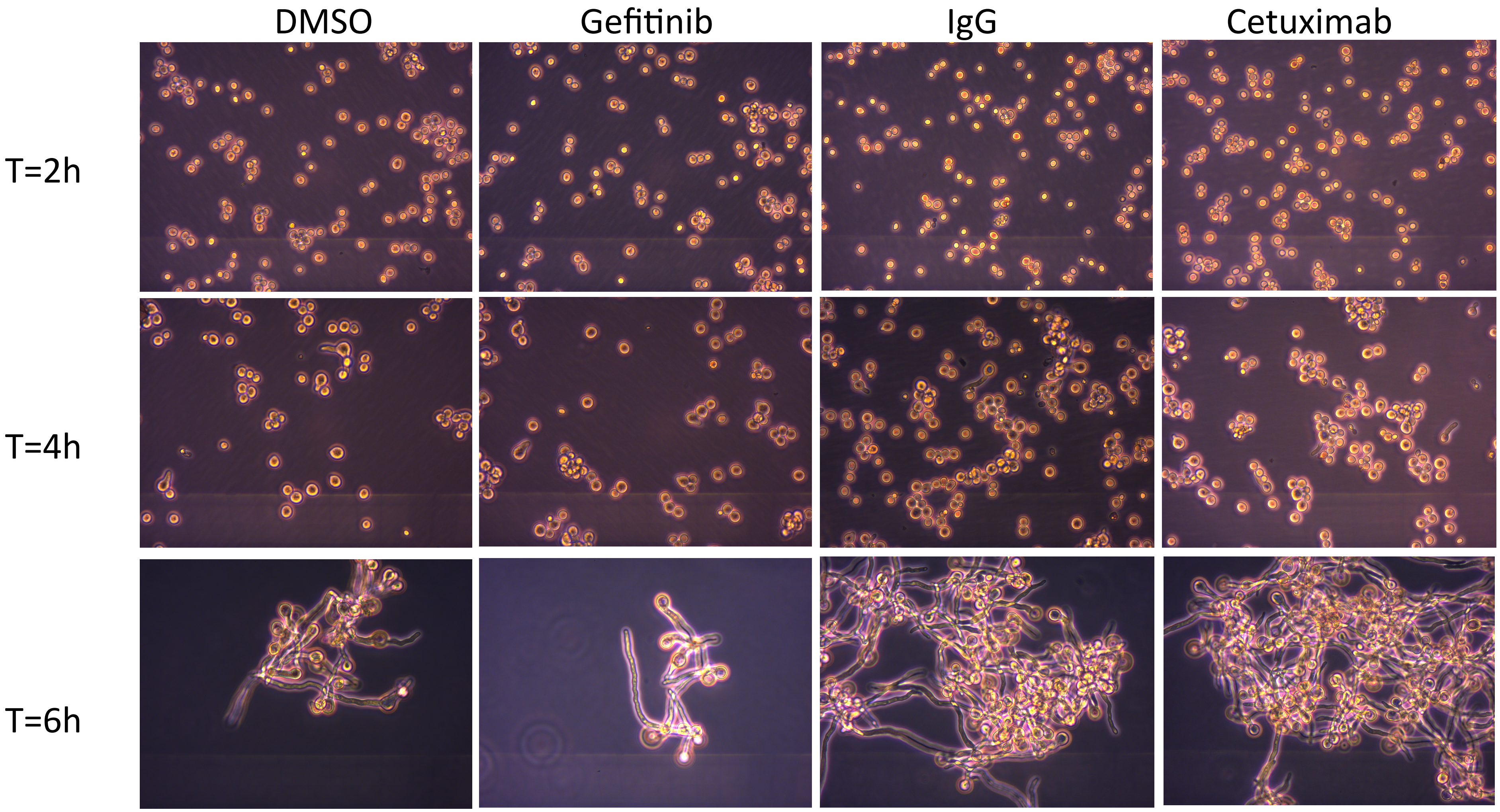

Supplementary Figure 2. EGFR inhibitors do not inhibit hyphal growth of *R. delemar*.
R. delemar spores were incubated in F12K media + 10% FBS in the presence of DMSO, 10 uM Gefitinib, 25 ug/ml IgG or 25 ug/ml Cetuximab in tissue culture dishes without shaking, 37°C, 5% CO_2.
