# Supplementary material for: Inhibition of EGFR Signaling Protects from Mucormycosis
Source: mBio. 2018 Aug 14;9(4):e01384-18. doi: 10.1128/mBio.01384-18 (PMC6094478; doi:10.1128/mBio.01384-18)
Supplement: FIG S3 [file mbo004184021sf3.pdf]

Supplementary Figure 3.

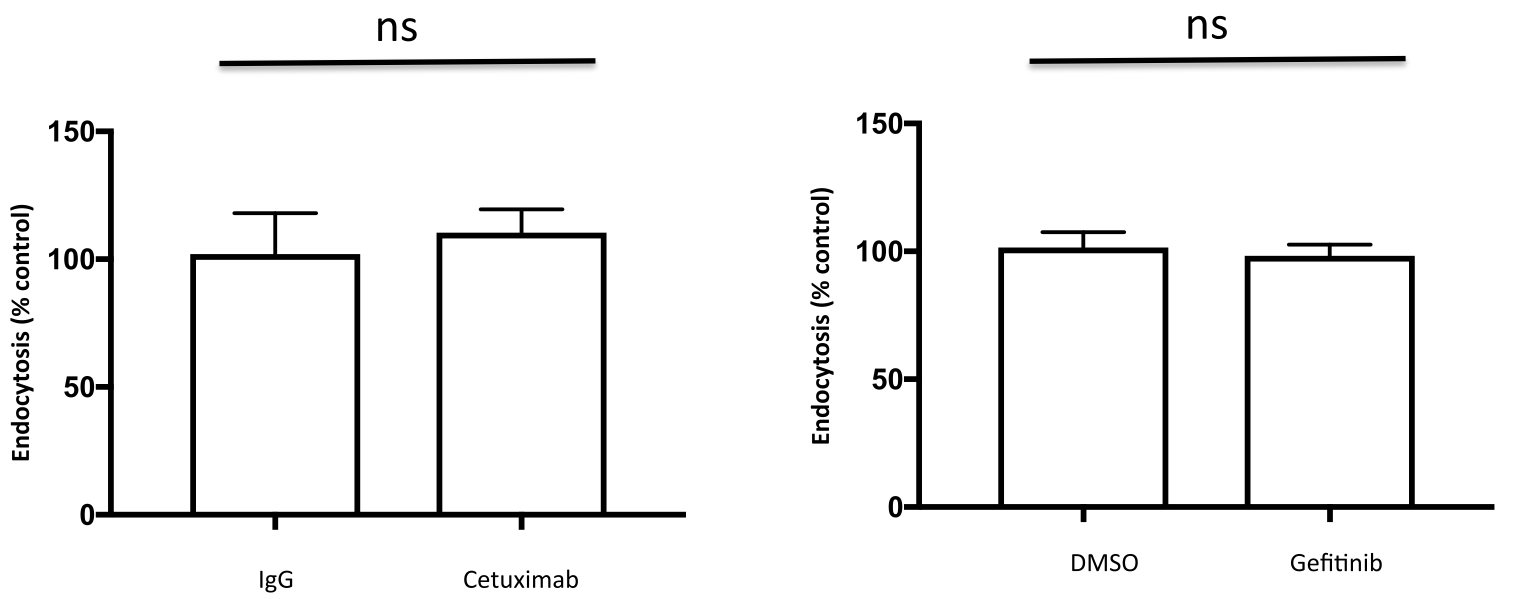

Supplementary Figure 3. Effects of *R. delemar* pretreatment with EGFR inhibitors on internalization of A549 cells. *R. delemar* spores were pre-treated with 25 μM Gefitinib or 25 μg/ml Cetuximab for 1h followed by washing with F12K + 10% FBS media. A549 alveolar epithelial cells were then infected with 2 x 10^5 *R. delemar* spores for 3h. Treatment vs control compared by Wilcoxon rank-sum test. Data are expressed as median ± interquartile range.
