# Supplementary material for: Inhibition of EGFR Signaling Protects from Mucormycosis
Source: mBio. 2018 Aug 14;9(4):e01384-18. doi: 10.1128/mBio.01384-18 (PMC6094478; doi:10.1128/mBio.01384-18)
Supplement: FIG S4 [file mbo004184021sf4.pdf]

Supplementary Figure 4.

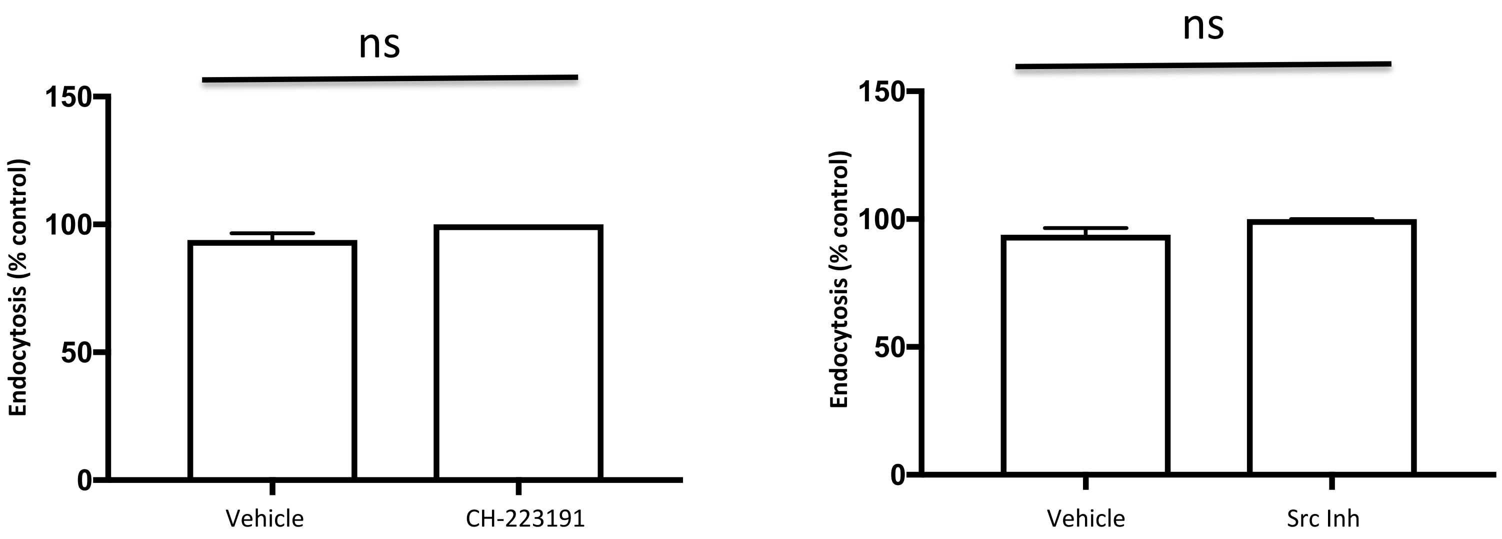

Supplementary Figure 4. Effects of Ahr and Src inhibition on *R. delemar* internalization and damage. A549 alveolar epithelial cells were pre-treated with 10 µM CH-223191 or 10 µM Src Inhibitor for 1 h followed by 3 h infection with 2 x 10^5 *R. delemar* spores that were germinated for 1 h. Control vs treatment *P< 0.05 by Wilcoxon rank-sum test. Data are expressed as median ± interquartile range.
